# PNPase knockout results in mtDNA loss and an altered metabolic gene expression program

**DOI:** 10.1371/journal.pone.0200925

**Published:** 2018-07-19

**Authors:** Eriko Shimada, Fasih M. Ahsan, Mahta Nili, Dian Huang, Sean Atamdede, Tara TeSlaa, Dana Case, Xiang Yu, Brian D. Gregory, Benjamin J. Perrin, Carla M. Koehler, Michael A. Teitell

**Affiliations:** 1 Molecular Biology Institute Interdepartmental Program, University of California Los Angeles, Los Angeles, California, United States of America; 2 Department of Pathology and Laboratory Medicine, University of California Los Angeles, Los Angeles, California, United States of America; 3 Department of Bioengineering, University of California Los Angeles, Los Angeles, California, United States of America; 4 Department of Chemistry and Biochemistry, University of California Los Angeles, Los Angeles, California, United States of America; 5 Department of Biology, University of Pennsylvania, Philadelphia, Pennsylvania, United States of America; 6 Department of Biology, Indiana University–Purdue University Indianapolis, Indianapolis, Indiana, United States of America; 7 Jonsson Comprehensive Cancer Center, University of California Los Angeles, Los Angeles, California, United States of America; 8 Broad Center for Regenerative Medicine and Stem Cell Research, University of California Los Angeles, Los Angeles, California, United States of America; 9 Department of Pediatrics, University of California Los Angeles, Los Angeles, California, United States of America; 10 California NanoSystems Institute, University of California Los Angeles, Los Angeles, California, United States of America; Instituto de Investigacion Hospital 12 de Octubre, SPAIN

## Abstract

Polynucleotide phosphorylase (PNPase) is an essential mitochondria-localized exoribonuclease implicated in multiple biological processes and human disorders. To reveal role(s) for PNPase in mitochondria, we established PNPase knockout (PKO) systems by first shifting culture conditions to enable cell growth with defective respiration. Interestingly, PKO established in mouse embryonic fibroblasts (MEFs) resulted in the loss of mitochondrial DNA (mtDNA). The transcriptional profile of PKO cells was similar to rho^0^ mtDNA deleted cells, with perturbations in cholesterol (FDR = 6.35 x 10^−13^), lipid (FDR = 3.21 x 10^−11^), and secondary alcohol (FDR = 1.04x10^-12^) metabolic pathway gene expression compared to wild type parental (TM6) MEFs. Transcriptome analysis indicates processes related to axonogenesis (FDR = 4.49 x 10^−3^), axon development (FDR = 4.74 x 10^−3^), and axonal guidance (FDR = 4.74 x 10^−3^) were overrepresented in PKO cells, consistent with previous studies detailing causative PNPase mutations in delayed myelination, hearing loss, encephalomyopathy, and chorioretinal defects in humans. Overrepresentation analysis revealed alterations in metabolic pathways in both PKO and rho^0^ cells. Therefore, we assessed the correlation of genes implicated in cell cycle progression and total metabolism and observed a strong positive correlation between PKO cells and rho^0^ MEFs compared to TM6 MEFs. We quantified the normalized biomass accumulation rate of PKO clones at 1.7% (SD ± 2.0%) and 2.4% (SD ± 1.6%) per hour, which was lower than TM6 cells at 3.3% (SD ± 3.5%) per hour. Furthermore, PKO in mouse inner ear hair cells caused progressive hearing loss that parallels human familial hearing loss previously linked to mutations in PNPase. Combined, our study reports that knockout of a mitochondrial nuclease results in mtDNA loss and suggests that mtDNA maintenance could provide a unifying connection for the large number of biological activities reported for PNPase.

## Introduction

Polynucleotide phosphorylase (PNPase) is a conserved 3’-5’ exoribonuclease that bacteria and most eukarya express, but is absent in archae [1, 2]. In addition to phosphorolytic RNA degrading activity, bacterial PNPase catalyzes template independent polymerization of RNA [3, 4]. The enzymatic features of bacterial PNPase have been well studied [4–10] and recent discoveries reveal bacterial PNPase involvement in modulating levels of multiple mRNAs and sRNAs [4, 11–13], an etiology in cold-shock [14–16] and oxidative stress responses [17], biofilm formation [18–20], virulence [21], and even DNA recombination, repair and mutagenesis [22–25].

Similar to its bacterial counterpart, mammalian PNPase has several roles in RNA homeostasis, and it has also been found to function within mitochondria. Constitutive PNPase knockout (PKO) in mice is lethal at embryonic day 8, because PNPase is essential for maintaining mitochondrial homeostasis in all cell types [26, 27]. Mammalian PNPase exhibits enzymatic features that are similar to bacterial PNPase with a different optimal phosphate concentration for RNA degradation [28, 29]. PNPase localizes to both the intermembrane space (IMS) and matrix compartments of mitochondria, as revealed in several model systems and by multiple localization methods [26, 27, 30, 31]. Knockdown studies have shown variable effects on the processing and polyadenylation of mitochondrial RNA (mtRNA) that is transcribed from mitochondrial DNA (mtDNA) [32–34]. In addition to RNASET2, which is a mitochondrial RNA degrading enzyme [35], recent studies have established that PNPase and the hSUV3 RNA helicase form a mtRNA degrading complex and degrade mirror-mtRNAs, which are noncoding mtRNAs that are antisense and complementary to coding mtRNAs [31, 34, 36].

Also similar to bacterial PNPase, mammalian PNPase participates in many physiologic pathways beyond mtRNA regulation. One study suggests that PNPase regulates c-Myc levels possibly through interactions with EGFR [37], whereas another study suggests PNPase controls an interferon-β (IFNβ)-induced reduction of c-Myc mRNA [38]. PNPase modulates an IFNβ-induced decrease of mir-221 levels that results in growth inhibition in melanoma cells [39] and it regulates nucleus encoded small non-coding RNA import into mitochondria [27, 40]. PNPase is a type-I IFN induced gene [41] and over-expression at supra-physiologic levels affects reactive oxygen species (ROS) generation and NF-κB activation [42]. Recently, PNPase was linked to metabolism control during somatic cell reprogramming to induced pluripotent stem cells [43, 44]. In humans, PNPase mutations genetically link to hereditary hearing loss, encephalomyopathy, and axonal and auditory neuropathy, gut disturbances, chorioretinal defects, Leigh syndrome, and delayed myelination [45–50]. Combined, PNPase affects many essential cellular processes and pathways that regulate organism physiology and pathology without a unifying theme or underlying mechanism, which requires further investigation.

Almost all investigations to date elevate PNPase to supra-physiologic levels in gain-of-function studies or use knockdown approaches with incomplete loss of PNPase in loss-of-function work to gain insight into mammalian PNPase activities. A few studies examined changes in gene expression profiles with gain and loss of PNPase function using these approaches to help explain the plethora of impacted physiologic systems [51, 52]. However, no study eliminated PNPase completely to evaluate the impact on global gene expression and cell function, likely due to the essential role of PNPase for cell growth and survival under usual conditions *in vivo* and in culture. Given its mitochondrial localization, roles in mtRNA regulation, and its activity in small nucleus-encoded RNA import into mitochondria to control respiration, we reasoned that reducing the dependence of cells on mitochondrial function first could generate a suitable internal cell environment for stable knockout of PNPase. Here, we established complete PKO cells by first reducing immortalized mouse embryonic fibroblast (MEF) dependence on respiration. The resulting PKO cell lines enabled studies of changes in gene expression and cell function directly due to PNPase loss. We also examined the effect of PNPase loss-of-function in inner ear hair cells in mice, as homozygous PNPase mutation or loss links with familial hearing loss in humans [45].

## Results

### PNPase knockout results in loss of mtDNA and inability to respire

**Fig 1:**
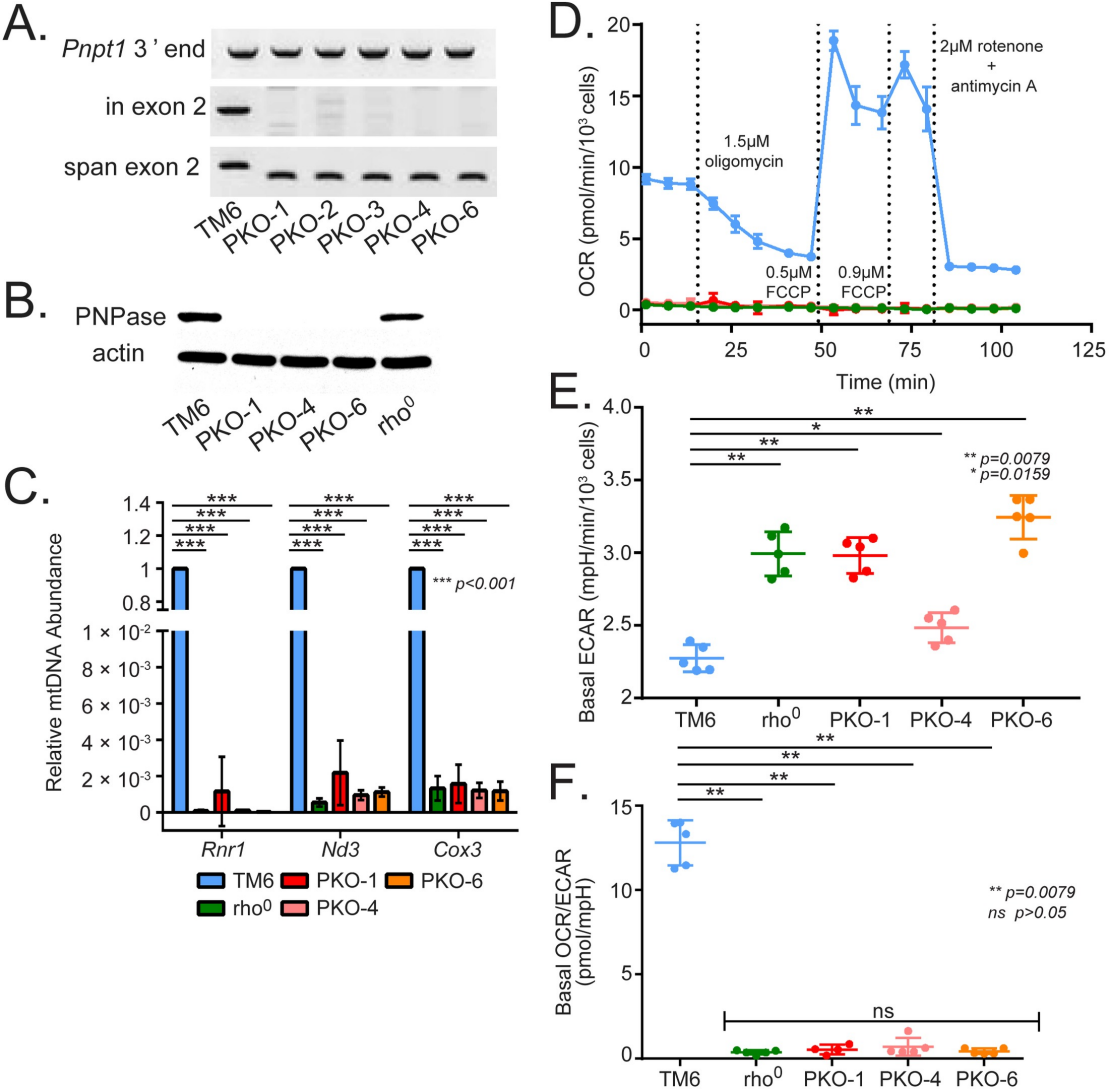
PKO results in loss of mtDNA. (A) PCR to evaluate *Pnpt1* (encoding PNPase) exon 2 deletion in the parental MEF line, TM6, and 5 independent PKO lines (n = 5). Three primer sets include one to examine *Pnpt1* exon 2 (in exon 2), a flanking set to capture deletion of exon 2 (span exon 2), and a control set that amplified the 3’ end of *Pnpt1* (PNPase 3’ end). (B) Representative immunoblot for PNPase protein in TM6, rho^0^ and 3 PKO clones. Total protein lysates were separated by SDS-PAGE and analyzed using polyclonal antibodies that target PNPase and β-actin. (C) TaqMan quantitative PCR (qPCR) analysis of a control nucleus encoded *Tfrc* gene and the mtDNA encoded *Rnr1*, *Nd3*, *and Cox3* genes in TM6 and 3 PKO cell lines. This experiment was performed in biological triplicates (n = 3, ± SD). (D-F) Respiration analysis using a Seahorse XFe96 Extracellular Flux Analyzer on the cell lines in (B). These experiments were performed in biological quintuplicates, normalized to cell number per 10^3^ cells. (D) Oxygen consumption rate (OCR) during mitochondrial stress, (E) basal extracellular acidification rate (basal ECAR), and (F) basal OCR/ECAR ratios are shown (n = 5, ± SD).

Our previous attempts to generate a PKO MEF line failed because deletion of the PNPase-encoding gene, *Pnpt1*, ultimately caused cell death [27]. Given its essential role in maintaining mitochondrial homeostasis and its mitochondrial localization, we considered that PKO might disrupt mitochondrial functions required for survival [26]. To test this idea, and because cell lines are known to rely on mitochondria to varying extents, we sought to establish PKO MEFs using a two-step approach. First, we generated a cell line that lacked mtDNA (rho^0^) using *Pnpt1*^*fl/fl*^ MEFs isolated from our *Pnpt1*^*fl/fl*^ mouse colony. SV-40 large T-antigen immortalized *Pnpt1*^*fl/fl*^ MEFs, designated TM6, were incubated in media containing ditercalinium chloride and uridine for 3 weeks to eliminate the mtDNA [53]. PCR validated, respiration defective rho^0^ *Pnpt1*^*fl/fl*^ MEFs were then infected with an adenovirus expressing Cre recombinase to delete the *loxP*-flanked portion of exon 2 in *Pnpt1*, generating non-functional PNPase-encoding alleles that translate out-of-frame with multiple stop codons in exons 3, 4, and 6 [27]. Using this strategy, we obtained numerous independent rho^0^ PKO MEF clones, indicating that eliminating mtDNA first provides a permissive internal cell environment for the complete loss of PNPase (data not shown).

rho^0^ mammalian cells are pyrimidine auxotrophs that require uridine media supplementation to grow due to an inactive dihydroorotate dehydrogenase enzyme in the mitochondrial inner membrane, resulting from a non-functional electron transport chain (ETC) [54]. Encouraged by the generation of a PKO in rho^0^ MEFs, we considered whether a simple pre-conditioning of mtDNA-containing MEFs with rho^0^ permissive, uridine-supplemented media, rather than by chemical removal of mtDNA, would result in PKO MEF lines. Therefore, we incubated *Pnpt1*^*fl/fl*^ TM6 MEFs in uridine-containing media for 3 weeks, followed by infection with an adenovirus expressing Cre recombinase. Multiple individual PKO MEF clones emerged from a background of dead and dying cells; these clones were isolated, expanded, and 5 clones were PCR verified for complete loss of *loxP*-flanked exon 2 in the *Pnpt1* gene, as performed previously using internal and external PCR primer sets (Fig 1A) [27]. We selected 3 PKO MEF clones, designated PKO-1, PKO-4, and PKO-6, for additional analyses. Immunoblotting revealed undetectable PNPase protein in these 3 PKO clone lines, in contrast with strong PNPase expression from *Pnpt1*^*fl/fl*^ TM6 and rho^0^ MEFs (Fig 1B). Thus, mtDNA-containing MEFs conditioned to grow in uridine-supplemented media tolerate PKO, whereas MEFs in standard growth media are non-permissive for PNPase loss.

To examine the impact of PKO on cell function, we first determined the effects on mtDNA content because chemically induced loss of mtDNA enabled subsequent PKO. Surprisingly, the TaqMan qPCR assay using probes specific to *Rnr1*, *Nd3* and *Cox1* showed no signal for all 3 PKO clones, identical to the lack of mtDNA in rho^0^ MEFs (Fig 1C). This result suggested that PKO MEFs lost mtDNA. Additionally, PicoGreen double-stranded DNA dye staining using fluorescence microscopy also suggested that mtDNA was not present in PKO cells (S1 Fig). Whereas the TM6 MEF cells displayed green puncta in the cytosol marking abundant mtDNA, rho^0^ and PKO MEFs lacked green cytosolic puncta, confirming the loss of mtDNA. All 3 PKO clones and rho^0^ MEFs showed no mitochondrial respiration, which contrasted with TM6 MEFs, as measured by a Seahorse XFe96 extracellular flux analyzer (Fig 1D). This result was consistent with loss of mtDNA and resultant failure of the ETC (Fig 1C). Rather, these PKO cells manufacture energy by substantially increasing glycolysis and show an elevated basal extracellular acidification rate (ECAR) relative to TM6 MEFs (*P* value = 0.0079 for PKO-1, PKO-6, and rho^0^ to TM6 comparisons, P value = 0.0159 for PKO-4 to TM6 comparison) (Fig 1E). In cells with low or absent respiration, ECAR is a good approximation of glycolysis and lactate excretion. A plot of the ratio of basal OCR/ECAR provides a remarkable graphic display for the metabolic alteration caused by PKO (*P* value < 0.0079 for all PKO and rho^0^ to TM6 comparisons) (Fig 1F). Combined, these genetic, protein, and functional data indicate that MEFs that lose PNPase cannot respire or maintain the mitochondrial genome.

### Transcriptional, growth and cell cycle profiles are similar for PKO and rho^0^ cells

**Fig 2:**
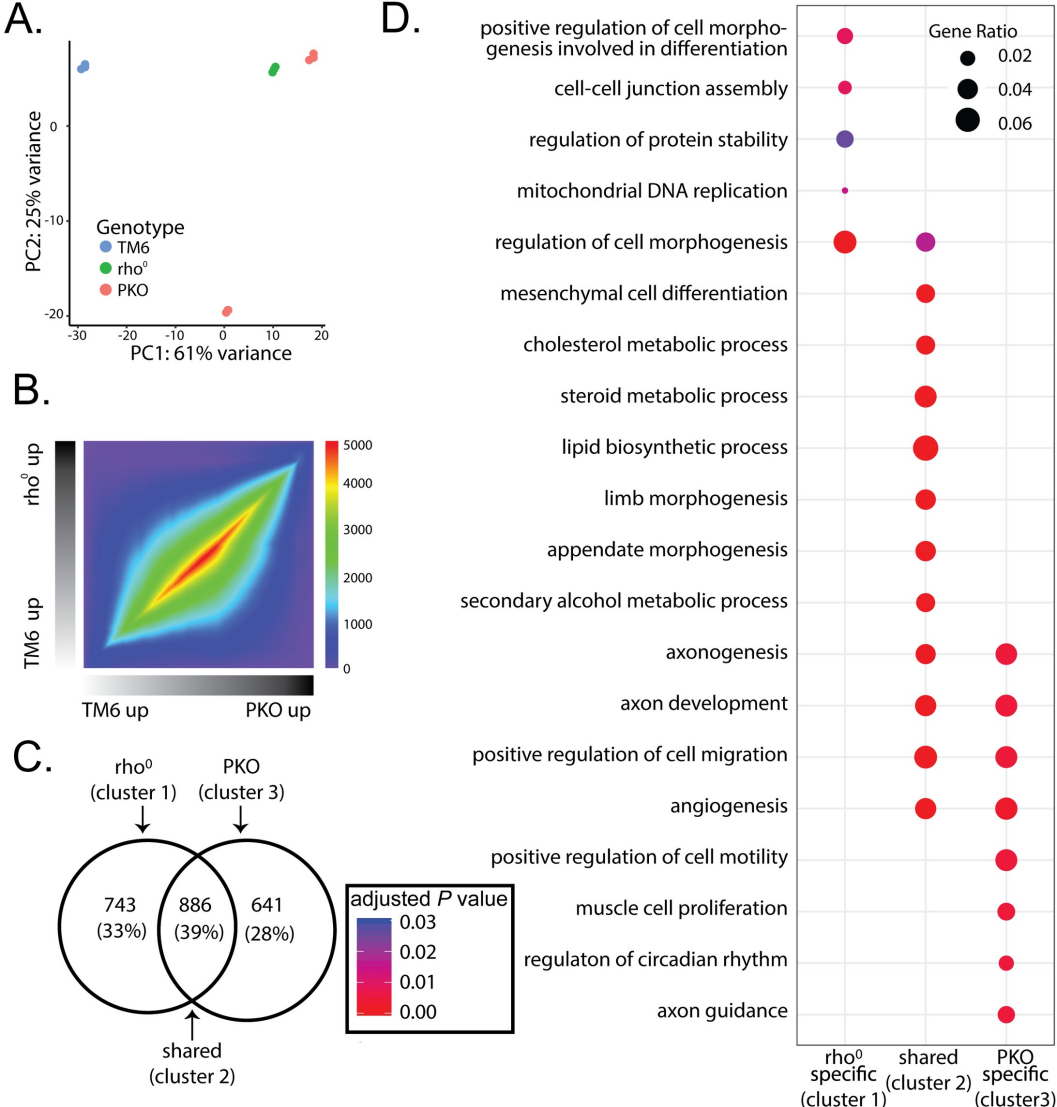
PKO cell gene expression patterns converge and diverge with rho^0^ MEFs. (A) Principal Components Analysis (PCA) of TM6, rho^0^, PKO-1, and PKO-6 MEFs (n = 3 biological replicates per line; 12 replicates total). Expression profiles for each sample were plotted for their PC1 and PC2 scores, providing primary and secondary sources of variance, respectively. Samples were color-coded based on cell type. (B) Rank-rank hypergeometric overlap (RRHO) maps of the log_2_ fold-change expression profiles between PKO and rho^0^ MEFs with respect to TM6 MEFs. Color coding represents–log_10_ transformed hypergeometric *P* values, with higher values indicating strength of overlapping enrichment between PKO and rho^0^ gene lists. (C) Venn diagram of differentially expressed genes (DEGs) between rho^0^ MEFs (left) and PKO clones (right) with respect to TM6 MEFs. DEGs were identified in each comparison using an absolute log_2_ fold-change threshold above 0.5 and a false discovery rate (FDR) adjusted Wald *P* value below 0.01. Clusters indicate rho^0^ specific (cluster 1), PKO specific (cluster 3), and shared DEGs (cluster 2). (D) Dotplot of Gene Ontology (GO) overrepresentation analysis (ORA) of the rho^0^ specific, shared, and PKO specific DEG clusters. Dot size indicates the ratio of genes from the selected ontology set in the DEG cluster, color indicates significance of gene set overrepresentation in the cluster (FDR adjusted *P* value) following hypergeometric significance testing.

To determine the effect of PNPase loss on gene expression and the potential to alter cell physiology, we performed RNA-Seq analyses on TM6 MEFs, rho^0^ cells derived from TM6 MEFs and PKO-1 and PKO-6 MEFs. PKO-4 cells were excluded from analysis because of aneuploidy detected by cell cycle profiling (data not shown). Principal components analysis (PCA), used to determine sample covariance with respect to their transcriptomic features, indicated extensive transcriptional variance, denoted by the first principal component (PC1), between TM6 MEF cells and the remaining cell lines (Fig 2A). Isolated PKO cells clustered away from the parental TM6 cell line and showed variability in inter-clone gene expression with little intra-clone variation (Fig 2A). However, PKO-1 and PKO-6 MEFs displayed similar expression signatures to rho^0^ TM6 MEFs (Fig 2A). A rank-rank hypergeometric overlap (RRHO) analysis calculating the significance between overlaps of averaged PKO and rho^0^ expression changes with respect to TM6 indicates strong correlative overlap in expression profiles between PKO and rho^0^ cells (maximum–log_10_(*P* value) > 4500) (Fig 2B). We identified a significant overlap in up- and down-regulated transcripts within PKO and rho^0^ signatures and additionally sought to identify differentially expressed genes (DEGs) in both expression profiles. Our analysis identified 1,629 DEGs between rho^0^ and TM6 MEFs, with a false discovery rate (FDR) adjusted *P* value below 0.01 and an absolute log_2_ fold-change above 0.5, and 1,527 DEGs between PKO and TM6 MEFs using the same thresholds (Fig 2C). Of the DEGs expressed by rho^0^ and PKO MEFs, 886 genes were in common, as anticipated due to significant overlaps in RRHO analysis and similar cellular physiology with the loss of mtDNA (Fig 2C).

We performed Gene Ontology (GO) gene set overrepresentation analysis (ORA) for each area represented in the Venn diagram (Fig 2C) and examined DEGs that are specific to rho^0^ MEFs (cluster 1) and PKO MEFs (cluster 3) and those DEGs shared between rho^0^ and PKO MEFs (cluster 2). GO terms overrepresented in cluster 1 identified processes including regulation of cell morphogenesis (FDR = 4.45 x 10^−4^), cell-cell junction assembly (FDR = 8.70 x 10^−3^), mitochondrial DNA replication (FDR = 0.014), and regulation of protein stability (FDR = 0.026) (Fig 2D). Genes whose expression associates with mtDNA replication are elevated in rho^0^ and reduced in TM6 MEFs (S2 Fig). These genes include a subunit of ribonucleotide reductase that regulates cytosolic nucleotide pools (*Rrm2b*), an RNase that removes an RNA primer in replicating mtDNA (*Rnaseh1*), and a pyrimidine transporter (*Slc25a33*) (S2 Fig).

Amongst pathways overrepresented by PKO and rho^0^ MEF DEGs (cluster 2) are cholesterol metabolic processes (FDR = 6.35 x 10^−13^), sterol metabolic processes (FDR = 6.35 x 10^−13^), lipid biosynthetic processes (FDR = 3.21 x 10^−11^), and secondary alcohol synthesis (FDR = 1.04 x 10^−12^), which were all reduced in both cell types (S2 Fig). Cholesterol and sterol biosynthetic process repression is consistent with data from PNPase shRNA knockdown in melanoma cell lines, reported previously [52].

**Fig 3:**
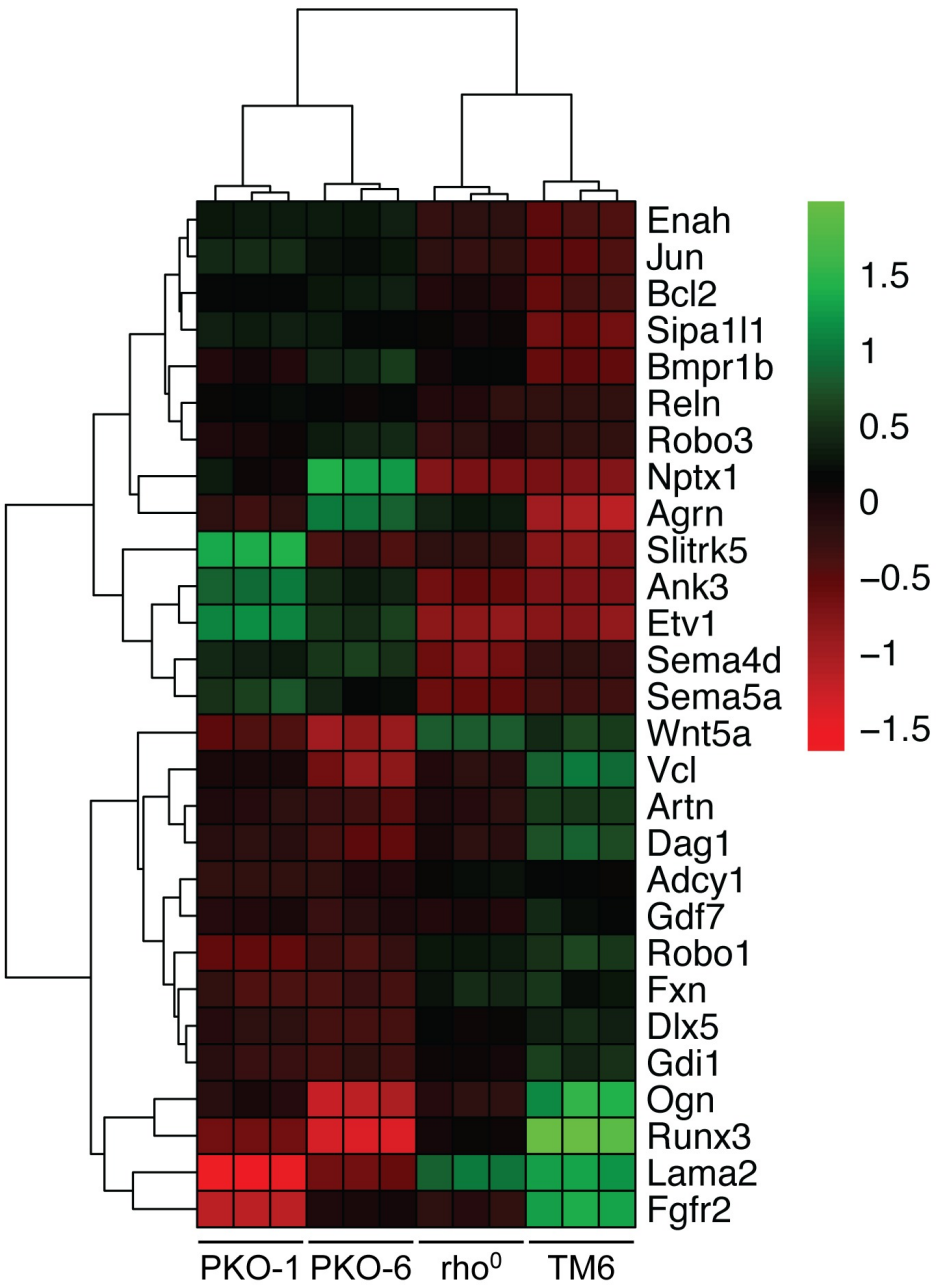
Heatmap of overrepresented neuronal function pathways in DEG cluster 3. Axonogenesis (GO:0007409), axon guidance (GO:0007411), and axon development (GO:0061564) genes overrepresented in cluster 3. Heatmap is hierarchically clustered based on Euclidean distance and Ward linkage. Heatmap values are plotted as the *variance stabilized transform* (VST) subtracted by the gene row average mean of samples. (n = 3 biological replicates, 12 total).

Pathways pertaining to neuronal function are overrepresented in PKO specific cluster 3 and include axonogenesis (FDR = 4.49 x 10^−3^), axon development (FDR = 4.74 x 10^−3^), and axon guidance (FDR = 4.74 x 10^−3^) (Fig 2D). We extracted specific genes belonging to the axonogenesis, axon development and axon guidance pathways identified in the ORA for analysis (Fig 3A and S1 Table). Among this list are genes involved in survival of neural cells such as *Bcl2* and *Artn* [55, 56], cell junction or adhesion such as *Vcl* and *Agrn* [57, 58], and cell migration such as *Reln*, *Robo3*, *Sema4d*, *Sema5*, *Wnt5a*, *Dag1*, *Robo1*, and *Fgfr2* [59–66]. Although this study uses MEFs, it is interesting to note that the change in these particular genes correlate with the lack of PNPase expression relative to their TM6 and rho^0^ MEF counterparts, since PNPase mutations are linked to delayed myelination, hearing loss, encephalomyopathy, gut disturbances, and chorioretinal defects in humans [45–50]. Axonogenesis (FDR = 1.14 x 10^−3^) and axon development (FDR = 2.00 x 10^−4^) pathways are also overrepresented in genes shared between rho^0^ and PKO MEF DEGs (cluster 2). However, these axonogenesis and axon development pathway overrepresented genes (cluster 2) differ from genes in PKO MEF-specific cluster 3 for the same ontologies. Thus, both loss of PNPase and loss of mtDNA may contribute uniquely to neurologic pathologies in patients with PNPase mutations.

**Fig 4:**
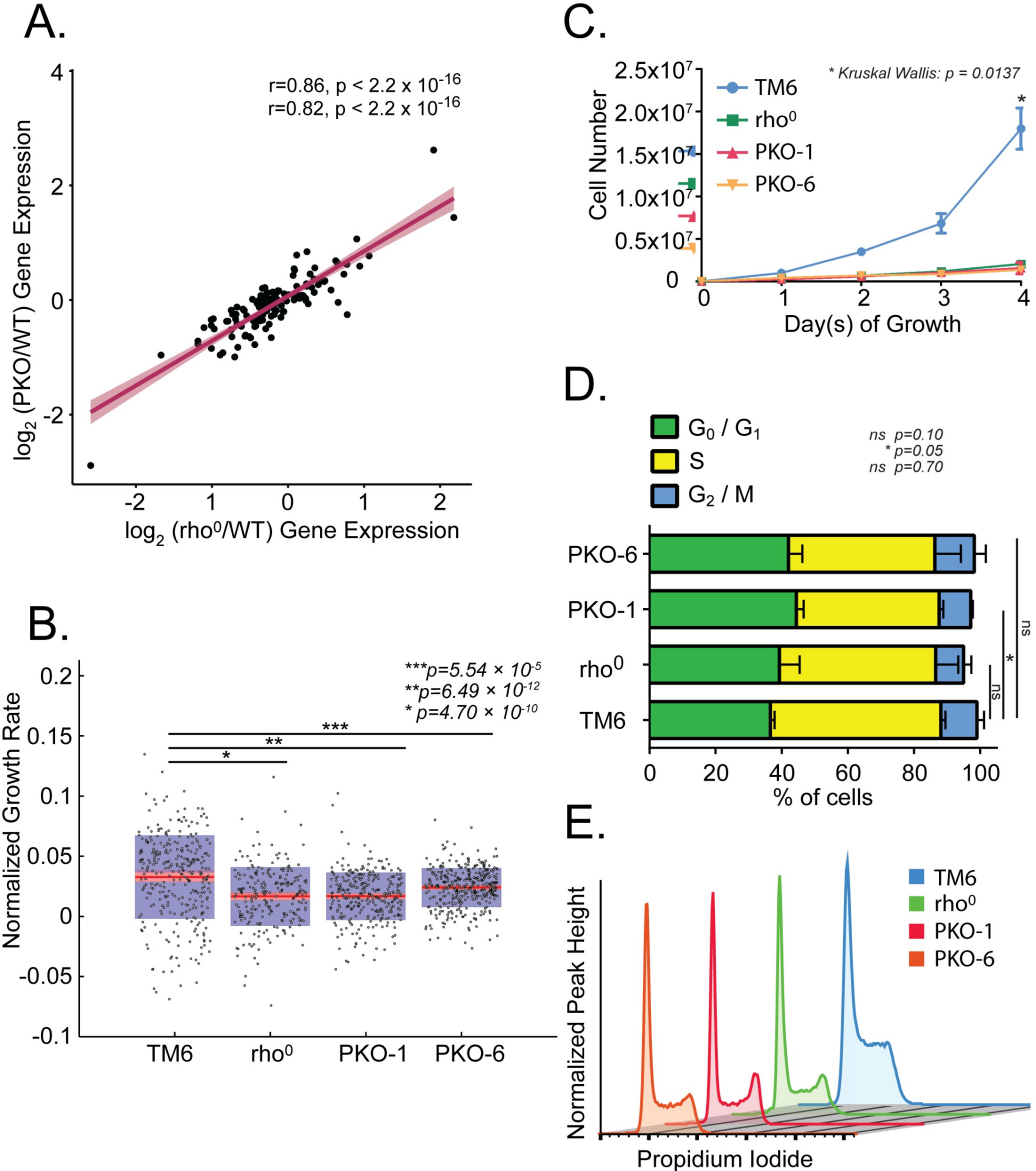
PKO and rho^0^ MEFs have similar cell growth, cell cycle and metabolic gene expression profiles. (A) Scatterplot of mitotic gene expression changes between PKO (y-axis) and rho^0^ (x-axis) MEFs with respect to TM6 MEFs (calculated as log_2_ fold-change) (n = 3 biological replicates per line, 12 total). Linear regression lines were fit and Pearson (top value) and Spearman (bottom value) correlation coefficients were calculated with accompanying *P* values calculated using two-tailed *t* significance tests. Gene sets were derived from KEGG database cell cycle ID MMU04110. (B) Growth (biomass accumulation) rates for TM6, rho^0^, PKO-1, and PKO-6 cells were quantified by live cell interferometry (TM6 n = 308, rho^0:^ n = 233, PKO-1: n = 303 PKO-6: n = 364). (Median = red stripe, 95% confidence interval = pink region ± SD = purple region. (C) Proliferation of TM6, rho^0^, PKO-1 and PKO-6 MEFs by manual cell counting (n = 3, ± SD). (D) Stacked barplots of phases of the cell cycle in percentage distributions for TM6, rho^0^, PKO-1, and PKO-6 MEFs (n = 3, ± SD). (E) Representative flow cytometry profiles for TM6, rho^0^, PKO-1, and PKO-6 MEFs. DNA was stained with propidium iodide.

Because of the concordance in gene expression profiles between PKO and rho^0^ MEFs (Fig 2B), we examined cell growth, cell cycle progression, and metabolic features in these cell types. Genes implicated in mitotic progression (KEGG: MMU04110) show a strong positive correlation for rho^0^ and PKO relative to TM6 MEFs (Pearson Correlation Coefficient (PCC) = 0.86, *P* value < 2.2 x 10^−16^) (Fig 4A). Furthermore, given the importance of metabolism in cell growth and a key role for cholesterol biosynthesis in cell cycle progression, we assessed the correlation of metabolic gene expression between PKO and rho^0^ MEFs [67, 68]. Total metabolic gene sets (KEGG: MMU01100) showed similar positive correlations for PKO and rho^0^ expression profiles relative to TM6 (PCC = 0.79, *P* value < 2.2 x 10^−16^) (S3 and S4 Figs). We next assessed whether these correlated expression profiles show phenotypic similarity by measuring growth, or biomass accumulation, rates for TM6, rho^0^, and PKO MEFs using a quantitative phase microscopy (QPM) technique called live cell interferometry (LCI) [69]. As anticipated, rho^0^ MEFs grew much slower than TM6 MEFs, and PKO clones accumulated biomass as slowly as rho^0^ MEFs (Fig 4B). LCI quantification revealed a mean growth rate for TM6 and rho^0^ MEFs of 3.3% (SD ± 3.5%) and 1.7% (SD ± 2.7%) of normalized cell biomass per hour, respectively, with a *P* value of 4.7 x 10^−10^ (Fig 4B). PKO-1 and PKO-6 showed a mean growth rate of 1.7% (SD ± 2.0%) and 2.4% (SD ± 1.6%) of normalized cell biomass per hour, respectively, and with *P* values compared to the TM6 growth rate of 6.49 x 10^−12^ and 5.54 x 10^−5^, respectively (Fig 4B). Furthermore, cell proliferation studies supported LCI quantified growth rate profiling data in that rho^0^, PKO-1 and PKO-6 cells replicated much slower than TM6 MEFs (Kruskal Wallis *P* value = 0.0137) (Fig 4C). Finally, cell cycle analysis by flow cytometry revealed a trend in which PKO and rho^0^ MEFs have a lower proportion of cells in S phase compared to TM6 MEFs (Fig 4D). The mean percentage of the cell population in S phase is 51.6% (SD ± 1.36%) for TM6 MEFs, 47.3% for rho^0^ MEFs (SD ± 6.80%), 43.1% (SD ± 1.25%) for PKO-1 cells, and 44.3% (SD ± 7.84%) for PKO-6 cells. The proportion of cells in S phase is significantly reduced in the PKO-1 clone relative to TM6 (*P* value = 0.05) and trending towards a decrease between PKO-6 clone and TM6 (*P* value = 0.10), supporting a decrease in S phase for PKO cells (Fig 4D). The difference in cell cycling characteristics is easier to appreciate in the flow cytometry profiles of PKO-1 and PKO-6 MEFs, which resemble rho^0^ MEFs, whereas the TM6 MEF flow profile has a distinct shape from the others (Fig 4E). Thus, integrated RNA-Seq, QPM, proliferation, and cell cycle analyses clearly show that PKO MEFs resemble rho^0^ MEFs that do not respire.

### PKO in inner ear hair cells causes progressive hearing loss

The PNPase E475G mutation results in hereditary hearing loss [45]. We therefore examined PKO for physiological relevance by examining auditory effects in vivo using *Pnpt1*^*fl/fl*^ *x Atoh1-Cre* expressing mice (*Atoh1*-Cre PKO mice). *Atoh1* encodes a transcription factor of the basic helix-loop-helix family involved in hair cell differentiation, and *Atoh1*-Cre expression in *Pnpt1*^*fl/fl*^ mice results in knockout of PNPase in the inner ear hair cells [70]. Auditory brainstem recordings of *Atoh1*-Cre PKO mice showed progressive hearing loss especially at higher frequencies. Up until 4 weeks of age both control and *Atoh1*-Cre PKO mice showed similar hearing capacities between 4 kHz and 16 kHz (S5 Fig). However, even at 3 weeks of age, the hearing capacity of *Atoh1*-Cre PKO mice above 16 kHz was reduced compared to control mice (S5 Fig). By week 4, the lowest level of hearing for 4 weeks of age *Atoh1*-Cre PKO mice in the high frequency range at 32 kHz was above 78 db in contrast to 39 db for 3 weeks of age control mice (*P* value = 0.0238) (S5 Fig). Scanning electron microscopy (SEM) images of inner ear hair cell stereocilia show loss of cilia (yellow arrow) and stereocilia fusions (red arrow) in the middle turn and base of the cochlea that are responsible for hearing in the middle and high frequencies, respectively (S5 Fig). Overall, hearing impairment observed in *Atoh1*-Cre PKO mice recapitulates the hearing loss observed in PNPase mutation harboring patients and emphasizes the association of PNPase with sensorineural defects.

## Discussion

We report that loss of PNPase, an RNA degrading enzyme that localizes in mitochondria, results in unanticipated depletion of the mitochondrial genome. Transcriptome profiling of PKO MEFs correlates significantly with rho^0^ MEFs and exhibits reduced expression of cholesterol and lipid biosynthesis genes compared to control wild-type TM6 MEFs. Cell growth, proliferation, and cell cycle features are similar between PKO and rho^0^ MEFs, suggesting a role for PNPase in maintaining mtDNA. We speculate that PNPase loss-related changes in gene expression programs pertaining to axon function could suggest a link with reported mutant PNPase neuronal disease phenotypes in humans, including familial hearing loss. Modeling PNPase loss in mouse inner ear hair cells leads to progressive auditory loss, reinforcing a potential connection uncovered from mining gene expression profiling data presented here.

Since PNPase does not belong to any of the previously identified classes of genes associated with mtDNA maintenance defects, it seems surprising that PKO results in mtDNA loss. Deletions and mutations of genes involved in mtDNA replication and repair (*POLG*, *TWNK*, *TFAM*, *RNASEH1*, *LIG3* and *MGME1*), cytosolic and mitochondrial nucleotide pool regulating and import genes (*TK2*, *DGUOK*, *SUCLA2*, *SUCLG1*, *ABAT*, *TYMP*, *RRM2B*, *AGK*, and *MPV17*), along with mitochondrial dynamics genes (*OPA1* and *FBXL4*), all result in mtDNA loss [71, 72]. Thus, it is intriguing to speculate on how PNPase may regulate the maintenance of mtDNA in a cell.

We hypothesize several potential reasons why lack of PNPase results in loss of mtDNA. Impaired import of small non-coding RNAs from the cytosol may reduce levels of nucleus-encoded *MRP* RNA that is required for mtDNA replication. Accumulation of mirror mtRNAs occurs in PNPase knockdown cells and in cells expressing dominant negative hSUV3, the binding partner of PNPase. Therefore, it is also possible that accumulation of mirror mtRNAs may somehow inhibit replication of mtDNA. Furthering this idea are studies that report that loss of hSUV3 in human cells and mSUV3 +/- haploinsufficiency in mice result in decreased mtDNA copy numbers and increased mtDNA mutations [31, 36, 73, 74]. PNPase functions in DNA repair in bacterial systems [24] and mammalian PNPase may have a similar function in mitochondria. We also cannot exclude that the loss of mtDNA may be a result of long-term dysregulated mtRNA metabolism. Although the mechanism(s) for how PNPase maintains mtDNA warrants further investigation, it nevertheless is interesting that PKO causes cells to functionally assume a rho^0^ phenotype.

We note that individuals with different mutations in *PNPT1* display a reduction in ETC function in specific tissues, yet their mtDNA is mostly retained [46, 47, 75, 76]. In particular, patient fibroblasts with heterozygous *PNPT1* c.227G>A; p.Gly76Asp and c.574C>T; p.Arg192* mutations [49] showed decreased oxygen consumption, and c.760C>A; p.Gln254Lys and c.1528G>C; p.Ala510Pro mutations have been linked to a decrease in *MT-CO1 and MT-CO2* encoded Complex IV protein levels [46]. Homozygous mutations of c.1160A>G; p.Gly387Arg also result in decreased Complex III and Complex IV activities in liver homogenates [47]. While these patients do present with severe respiratory chain defects, these observations do not compare with the abolishment of respiration in our PKO system (Fig 1D), where complete loss of mtDNA likely abrogates any capacity to establish a functional ETC for supporting cell respiration. Additionally, the minimal yet present levels of ETC protein and ATP production in several patients indirectly suggests that these patients still possess mtDNA [46, 76]. Unfortunately, only one study amongst the reported PNPase mutant patient analyses provide mtDNA level measurements. In homozygous *PNPT1* c.1160A>G; p.Gly387Arg patient derived fibroblasts, the mtDNA levels were decreased by 58% relative to the control, but this extent of decrease is not considered significant due to the large variability in control mtDNA levels [47]. PNPase protein expression levels vary amongst different PNPase mutations expressed in assessed patient tissues [45–48, 75, 76]. We suggest that even the minimal presence of mutant PNPase in these various patients may allow for a low amount of PNPase activity that can support the maintenance of mtDNA at reduced but functional levels. Therefore, the extent of mtDNA loss and ETC activity decrease is much more severe in the PKO MEF system compared to mutant PNPase patients, likely due to the complete loss of PNPase protein levels in PKO MEFs.

PNPase regulates oxidative phosphorylation during reprogramming of somatic cells to induced pluripotent stem cells and is an essential factor for maintaining mitochondrial homeostasis [26, 43, 44]. Understanding the specific functions of PNPase may reveal how RNA regulation may affect cellular metabolism. Here, we established PKO MEF lines utilizing uridine supplemented media and show that PKO cells lose their mtDNA. This brings a new perspective to PNPase activity and suggests that mtDNA levels are important to consider when dissecting PNPase functionality. Lastly, PNPase loss impairs neuron-relevant gene expression even in non-neuronal cell types and the Atoh1-Cre PKO mice show reduced hearing through auditory brainstem recording analysis. Altogether, our study describes an additional unsuspected function for PNPase in mtDNA maintenance, which could provide an underlying or unifying connection for its large number of reported activities in multiple biological systems and contexts.

## Materials and methods

### Cell culture and mice

*Pnpt1*^*fl/fl*^ MEFs, named TM6 [27], were grown at 37°C and 5% CO_2_ in Dulbecco’s Modified Eagle Medium supplemented with 10% Hyclone Fetal Bovine Serum (Thermo Scientific), 1X penicillin-streptomycin solution (Cellgro, Corning), and 50 μg/mL uridine (Sigma-Aldrich). MEF rho^0^ cells were established by culturing with 750 ng/mL ditercalinium dichloride dihydrochloride (NCI DCTD Developmental Therapeutics Program, NSC 335153, NIH), for 3 weeks followed by single cell colony isolation. PKO MEF lines were established by transduction of TM6 MEFs with a Cre-recombinase expressing adenovirus (SignaGen Laboratories cat # SL100707) in uridine- supplemented media, followed by isolation of single cell colonies. Atoh1-Cre PKO mice were established by crossing *Pnpt1*^*fl/fl*^ C57BL/6J mice with *Atoh1-Cre* transgenic C57BL/6J mice [70, 77]. All mice were housed in a pathogen-free animal facility at IUPUI, and the study was approved by the University of Minnesota Institutional Animal Care and Use Committee. Mouse experiments were designed and performed according to Animal Research: Reporting In Vivo Experiments (ARRIVE) guidelines, developed by the National Centre for the Replacement, Refinement & Reduction of Animals in Research (NC3Rs) guidelines.

### Characterization of PKO MEFs

Western blots were performed using standard molecular biology practices. PNPT 3370 antibody (Koehler/Teitell Labs) and β-tubulin MMS-410P (Covance) were used for immunoblotting. Assessments of mtDNA quantity were performed by TaqMan qPCR assay using mouse *Rnr1* probe (Thermo Scientific Mm04260177_s1), mouse *Nd3* probe (Thermo Scientific Mm04225292_g1), mouse *Cox1* probe (Thermo Scientific 4448484), mouse *Tfrc* probe (Thermo Scientific 4458366) and Amplitaq Gold 360 Master Mix (Thermo Scientific 4398881). DNA was extracted from proteinase K digested and RNase A treated samples through aqueous phase separation using phenol/chloroform/isoamyl alcohol. The resulting DNA was suspended in TE buffer, normalized to 20 ng/μL across samples, pooled, and serially diluted for standard curve generation in comparison to a no-template control. Biological triplicates of each sample were split into technical triplicates, and subsequently loaded on a Roche LightCycler 480 II (Roche 050152278001), with one preincubation cycle at 95°C for 10 min, followed by 45 amplification cycles at 95°C for 15s, to 60°C for 1 min. Raw cycle threshold (Ct) values were extracted for each well, averaged across technical replicates, and ΔCt values calculated for each experimental (*Rnr1*, *Nd3*, *Cox3*) and housekeeping gene (*Tfrc)*. ΔΔCt values were calculated for each pairwise condition replicate and 2^ΔΔCt^ transformed to generate an expression fold change relative to *Tfrc*. Expression fold changes per comparison were averaged and plotted using GraphPad Prism 6 (GraphPad Software, Inc.). Fluorescence microscopy images were obtained from cells treated with Mitotracker Red CMXRos (Thermo Scientific) and PicoGreen dye (Thermo Scientific). OCR and ECAR measurements were performed as previously described [78]. The following primers were used to check for the presence of *Pnpt1* exon 2 in the genome DNA: forward primer: TATCCTCTGGGAAACTGGCA, reverse primer: ATTCGTACTGCCCCAACAGG. The following primers were used to check for the deletion of exon 2 in *Pnpt1* that spans the exon 2 region: forward primer: TCGGGCACTCAGCTATTTGC, reverse primer: CACCAACGGCATGAATTGGG. The following primers were used to check for the presence of the 3’ end of *Pnpt1*: forward primer: ACCGCGACAATAACTGAAATC, reverse primer: GCAGCACTGCAGTCATGTTT.

### Seahorse extracellular flux analysis

Oxygen consumption rate (OCR) and extracellular acidification rate (ECAR) were measured as previously described [78]. We plated 20,000 cells/well in a XFe96 microplate (Seahorse Bioscience). Oligomycin (ATP synthase inhibitor) was injected at 1.5μM, FCCP (uncoupling agent) was injected at 0.5μM followed by a secondary injection of 0.9μM, and Antimycin A plus Rotenone (inhibitors of Complex III and I, respectively) were injected at 2μM each. Results were normalized per 10^3^ cells per well using the Operetta High Content Imaging System (PerkinElmer, Inc.).

### RNA extraction

TM6, rho^0^ MEFs, and PKO clones were grown in biological triplicates to 70–80% confluence and purified using TriZol Reagent (Life Technologies) or RNeasy Mini Kit (Qiagen). All samples used showed a A260/280 ratio > 2.00 (Nanodrop; Thermo Scientific). Prior to library preparation, RNA quality control was performed using the Advanced Analytical Technologies Fragment Analyzer (Advanced Analytical, Inc.) and PROSize 2.0.0.51 software. RNA Quality Numbers (RQNs) were computed per sample with a final score of 10.0 indicating fully intact total RNA per sample prior to library preparation.

### RNA-Seq library preparation

Strand-specific ribosomal RNA (rRNA) depleted RNA-Seq libraries were prepared from 1 **μg** of total RNA using the KAPA Stranded RNA-Seq Kit with Ribo-Erase (Kapa Biosystems, Roche). Briefly, rRNA was depleted from total RNA samples, the remaining RNA was heat fragmented, and strand-specific cDNA was synthesized using a first strand random priming and second strand dUTP incorporation approach. Fragments were then A-tailed, adapters were ligated, and libraries were amplified using high-fidelity PCR. All libraries were prepared in technical duplicates per sample (n = 12 samples, 24 libraries total), and resulting raw sequencing reads merged for downstream alignment and analysis. Libraries were paired-end sequenced at 2x125bp on an Illumina HiSeq.

### RNA-Seq pre-processing

PKO clones 1 and 6, rho^0^, and TM6 MEFs were each sequenced in biological triplicates (n = 3, 12 total samples). Raw sequencing reads were converted into fastq files and filtered for low quality reads and Illumina sequencing adapter contamination using bcl2fastq (Illumina). Trimmed reads were then aligned to the *Mus musculus* transcriptome, generated using the NCBI mm10 (NCBI/mm10/GRCm38, December 2011) genome and RefSeq gene annotation, using RSEM 1.2.25 prepare-reference (command parameters—bowtie—bowtie2—gtf $(BUILD)/genes.gtf $(GENOME).fa $(RSEM)) [79]. Transcript counts were estimated using RSEM 1.2.25 calculate-expression, and collapsed to gene level counts using RSEM 1.2.25 tbam2gbam [79]. Gene-level transcript counts were extracted from the results output using custom R/3.4.1 scripts.

### Differential gene expression analysis

The resulting sample gene count matrix was size factor normalized and analyzed for pairwise differential gene expression using R/3.4.2 Bioconductor 3.6 package DESeq2 v1.18.1. Expression changes were estimated using an empirical Bayes procedure to generate moderated fold change values [80, 81]. Significance testing was performed using the Wald test, and resulting *P* values were adjusted for multiple testing using the Benjamini-Hochberg procedure [82]. DEGs were filtered using an adjusted false discovery rate (FDR) *q* value < 0.01 and an absolute log_2_ transformed fold-change > 0.5. *Variance stabilized transform* (VST) values in the gene count matrix were calculated and plotted for principal component analysis (PCA) using DESeq2 [80, 81]. Scatterplots of gene expression fold-changes between TM6, rho^0^ and PKO MEFs were performed and Pearson/Spearman correlation coefficients calculated using R/3.4.1 package ggpubr v0.1.6 (https://cran.r-project.org/web/packages/ggpubr/index.html). Genes of interest were extracted and heat maps were prepared using R Bioconductor packages pheatmap v1.0.8 and gplots v3.0.1 [83, 84].

### Gene set overrepresentation analysis (ORA)

DEGs were extracted and analyzed for pathway/gene ontology (GO) term overrepresentation using the R/3.4.1 Bioconductor 3.6 package clusterProfiler v3.6.0 and ReactomePA v1.22.0, using a background gene set of all genes expressed with at least one read count in the sample gene count matrix [85, 86]. Overrepresented Reactome/KEGG pathways and GO terms were identified using significance testing cutoffs of *P* < 0.05, and an adjusted FDR < 0.25.

### Rank-rank hypergeometric overlap analysis (RRHO)

To determine overlapping significance between PKO and rho^0^ expression patterns relative to TM6, rank-rank hypergeometric overlap was performed using the online web server (http://systems.crump.ucla.edu/rankrank/rankranksimple.php)) [87]. Separate gene ranking lists were constructed according to the signed log_10_ transformed Wald test *q* value in fold change comparisons between PKO/TM6 and rho^0^/TM6. Hypergeometric testing was then performed to determine the significance of overlap between ranks in both datasets. Heat map values were plotted as the signed log_10_ transformed hypergeometric *P* value of overlap between ranks at the identified pixel; high values indicate enrichment of overlap, low values indicate reduced enrichment of overlap.

### Live cell interferometry (LCI)

Cells were plated into each well of an Ibidi 4 well Ph+ μ-slide (Ibidi, USA) at a density of 6 x10^3^ cells/cm^2^. 24 hours later, Ibidi anti-evaporation oil (Ibidi, USA) was added to seal the liquid opening of each well and loaded onto the LCI stage. All 4 wells were imaged continuously, every 10 minutes for 24 hours with 15–20 locations per well. The LCI set up consists of a Zeiss Axio Observer Z1 inverted microscope, an on-stage incubation chamber (Zeiss) with temperature, CO_2_ and humidity modulations. QPM images were captured by a SID4BIO (Phasics) quadriwave lateral shearing interferometry camera. All cells were imaged using a 20x 0.4NA objective and a 660nm collimated LED (Thorlabs) light source.

### Cell cycle flow cytometric analysis

Cells (~5 x 10^5^) were collected, washed once with 500 μl FACS buffer and resuspended in 200 μl hypotonic propidium iodide staining solution containing 10 mg/ml RNAse, 10% Triton X-100, 85 mg/ml trisodium citrate and 2 mg/ml PI (Roche). Data was obtained on FACS BD LSRII and FACS BD Fortessa flow cytometers (BD Biosciences). Cell cycle analysis was performed with FlowJo 10 software using the univariate model.

### Auditory brainstem response (ABR)

ABR waveforms were collected for frequencies between 4 kHz and 32 kHz at half-octave intervals, starting at supra-threshold levels and decreasing in 5 dB steps to a sub-threshold level. A Tucker-Davis Technologies System 3 was used to generate symmetrically shaped tone bursts 1 ms in duration with 300 μs raised cosine ramps that were delivered to a calibrated magnetic speaker. For this assay, n = 3 heterozygous control mice at 3 weeks (1 female, 2 male), n = 2 *Atoh1*-Cre PKO mice at 3 weeks (2 females) and n = 2 *Atoh1*-Cre PKO mice at 4 weeks (2 females) were analyzed. Mice were anesthetized with Avertin and scalp potentials were recorded with subdermal electrodes, with signals amplified 20,000 times, bandpass filtered between 0.03 and 10 kHz, digitized using a 20,000 kHz sampling rate and subjected to artifact rejection. Stacked waveforms were compared and the lowest level of stimulation that evoked an unambiguous ABR waveform was designated as the threshold.

### Scanning electron microscopy (SEM)

Mouse cochlea were dissected and fixed in 2.5% glutaraldehyde in 0.1 M sodium cacodylate buffer with 1 mM CaCl_2_ by perfusing dissected cochlea through the round and oval windows followed by incubation in the same solution at RT for 4 h. Following decalcification in 170 mM EDTA at 4°C for 16 h, the organ of Corti was dissected and processed for SEM as described [88]. Briefly, tissues were successively incubated in 2% each arginine, glycine, glutamic acid and sucrose in water, 2% each tannic acid and guanidine-HCl in water and then 1% osmium tetroxide. Samples were critical point dried from CO_2_ and sputter coated with platinum before viewing on a cold field emission SEM (Hitachi S4700). SEM images were taken using 1 male *Pnpt* ^*fl/+*^ *Cre*^*-*^ mouse at 6 weeks of age, and 2 female *Atoh1*-Cre PKO mice at 6 weeks of age. Mice were anesthetized with Avertin and euthanized via cervical dislocation.

### Statistical analysis

Statistical analysis performed for live cell interferometry and sequencing experiments are described in the above specified methods and figure legends. All other experiments were analyzed using GraphPad Prism 6.07 (GraphPad Software Inc.), reporting mean ± standard deviation (SD) for all conditions. *P* values reported for cell growth and ABR assays were calculated using a non-parametric Kruskal-Wallis ANOVA test. Pairwise *P* values reported for Seahorse extracellular flux and cell cycle distribution analyses were calculated using the non-parametric Mann-Whitney test. All significance testing was performed with a significance cutoff of *P* value < 0.05.

### Accession numbers

All raw RNA-Seq reads and processed gene count matrices were submitted to the NCBI Short Read Archive (SRA) and Gene Expression Omnibus (GEO), respectively, under GEO Accession number GSE111668.

## Supporting information

S1 Fig Immunofluorescence images of PKO cells show loss of mtDNA (related to Fig 1). Fluorescence microscopy of TM6, rho^0^, and representative PKO (PKO-4) MEF cell lines with PicoGreen staining for double-stranded DNA (left), MitoTracker Red (center), and an overlay (right).
(TIF)

S2 Fig Heatmaps of select overrepresented pathways in DEG clusters (related to Fig 2D). (A) Heat map of mtDNA replication genes (GO:0006264) overrepresented in cluster 1. (B) Cholesterol metabolic (GO:0008203), sterol metabolic (GO:0016125), lipid biosynthetic (GO:0008610), and secondary alcohol synthetic (GO:1902652) processes overrepresented in cluster 2.
(TIF)

S3 Fig PKO and rho^0^ MEFs show highly correlated metabolic gene expression profiles (related to Fig 4). Metabolic gene expression changes between PKO (y-axis) and rho^0^ (x-axis) MEFs with respect to TM6 MEFs (calculated as log_2_ fold-change) (n = 3 biological replicates per line, 12 total). Linear regression lines were fit and Pearson (top value) and Spearman (bottom value) correlation coefficients were calculated with accompanying *P* values calculated using two-tailed *t* significance tests. Gene sets were derived from KEGG database metabolic ID MMU00100.
(TIF)

S4 Fig PKO and rho^0^ MEFs show highly correlated gene expression profiles within specific metabolic pathways (related to Figs 3 and 4). Scatterplot of metabolic gene expression values between PKO (y-axis) and rho^0^ (x-axis) MEFs with respect to TM6 MEFs (calculated as log_2_ fold-change) (n = 3 biological replicates per line, 12 total). Linear regression lines were fit and Pearson (top value) and Spearman (bottom value) correlation coefficients were calculated with accompanying significance *P* values calculated using two-tailed *t* significance tests. Gene sets were derived from the KEGG database under the identification numbers indicated above each plot.
(TIF)

S5 Fig Loss of PNPase results in hearing loss. (A) Auditory brainstem response test for WT (black) (n = 3) and Atoh1-Cre PKO mice (red) at 3 weeks (n = 2) and 4 weeks (n = 2), error bars denotes standard error of mean. (B) SEM analysis of hair cell stereocilia (n = 2). Yellow arrows indicate regions that lack cilia, and red arrows indicate regions of stereocilia fusion.
(TIF)

S1 Table List of DEGs and overrepresented gene ontologies (related to Figs 2, 3, 4A, S2, S3 and S4). (A) List of DEGs identified between rho^0^ and TM6 MEFs. (B) List of DEGs identified between PKO and TM6 MEFs. (C) List of PKO-specific DEGs, shared DEGs, and rho^0^-specific DEGs. (D) Results of GO overrepresentation analysis (ORA) performed on DEG clusters in (C).
(XLSX)

## Abbreviations

DEG: differentially expressed gene
ETC: electron transport chain
FDR: false discovery rate
LCI: live cell interferometry
mtDNA: mitochondrial DNA
mtRNA: mitochondrial RNA
OXPHOS: oxidative phosphorylation
PCA: principal component analysis
PNPase: polynucleotide phosphorylase
PKO: PNPase knockout
RNA-Seq: RNA sequencing
GO: gene ontology
ORA: overrepresentation analysis
RT-PCR: reverse transcriptase-polymerase chain reaction
QPM: quantitative phase microscopy
SEM: scanning electron microscopy
MEF: mouse embryonic fibroblast
ECAR: extracellular acidification rate
OCR: oxygen consumption rate
