# Supplementary figures and images for: PNPase knockout results in mtDNA loss and an altered metabolic gene expression program
Source: PLoS One. 2018 Jul 19;13(7):e0200925. doi: 10.1371/journal.pone.0200925 (PMC6053217; doi:10.1371/journal.pone.0200925)

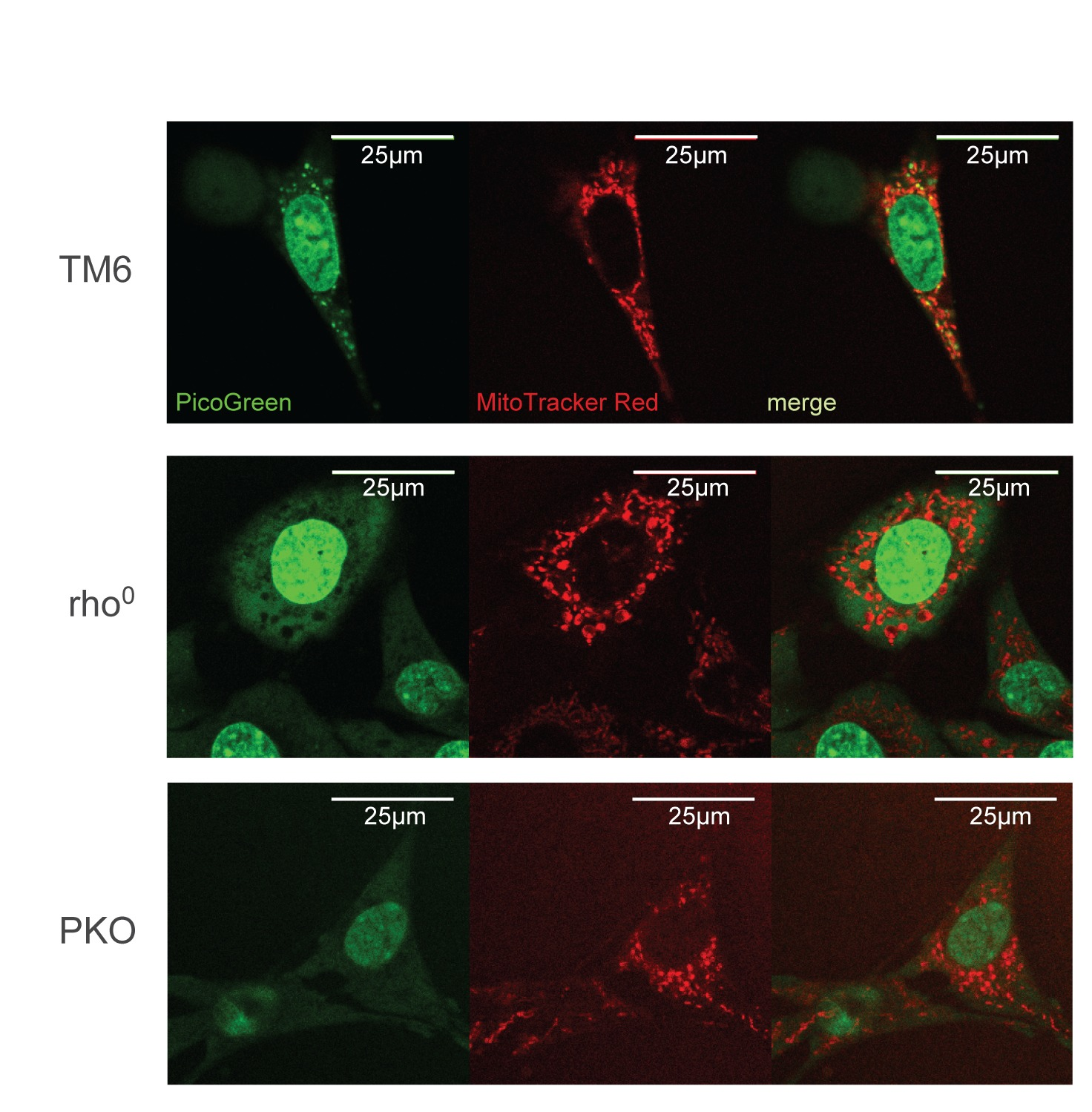

Supplement: S1 Fig — Fluorescence microscopy of TM6, rho0, and representative PKO (PKO-4) MEF cell lines with PicoGreen staining for double-stranded DNA (left), MitoTracker Red (center), and an overlay (right). (TIF) [file pone.0200925.s001.tif]

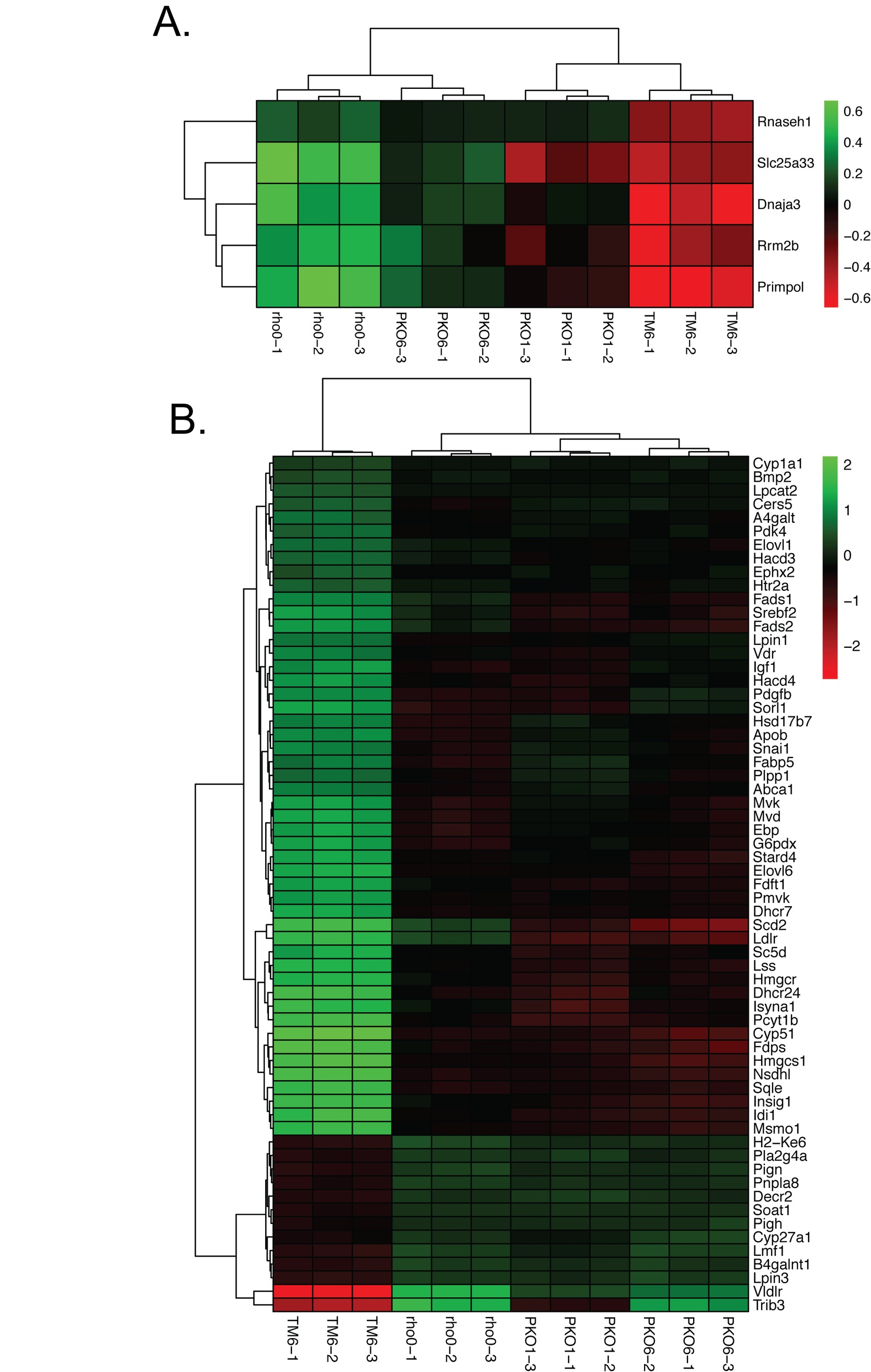

Supplement: S2 Fig — (A) Heat map of mtDNA replication genes (GO:0006264) overrepresented in cluster 1. (B) Cholesterol metabolic (GO:0008203), sterol metabolic (GO:0016125), lipid biosynthetic (GO:0008610), and secondary alcohol synthetic (GO:1902652) processes overrepresented in cluster 2. (TIF) [file pone.0200925.s002.tif]

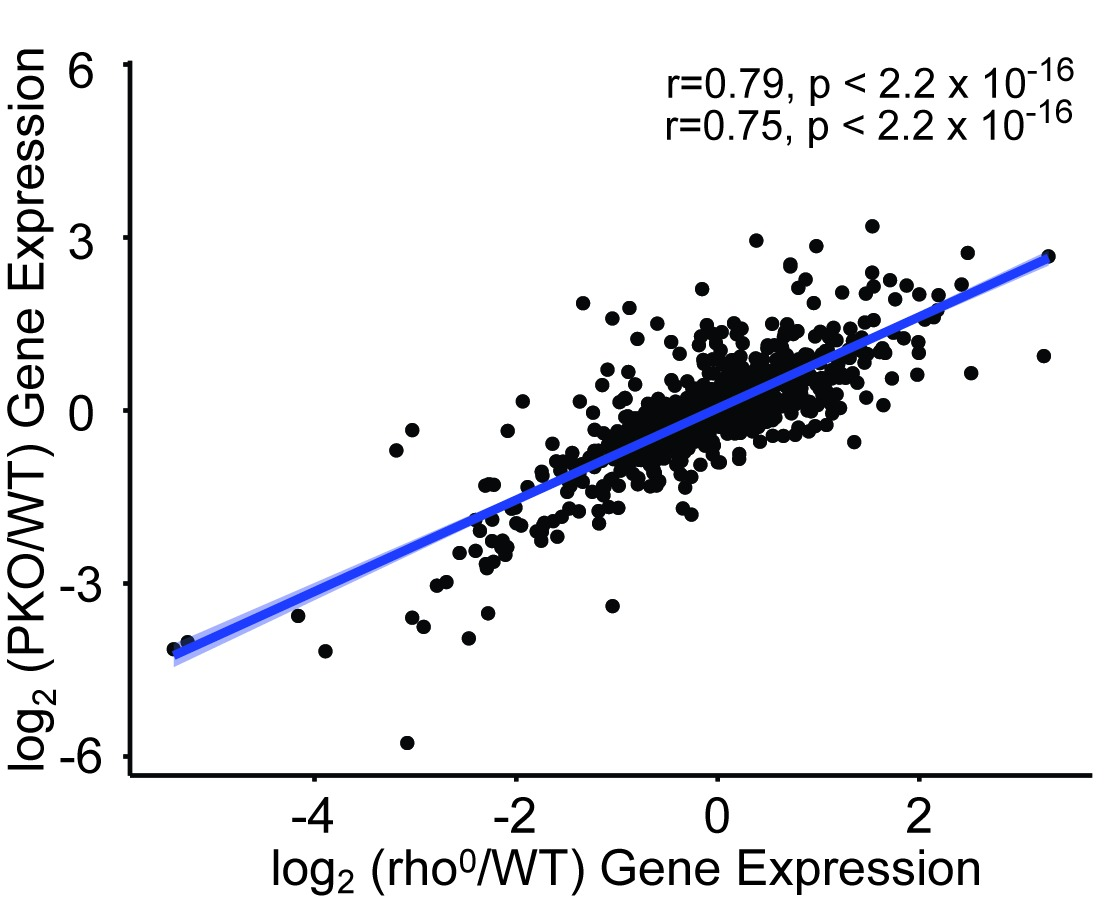

Supplement: S3 Fig — Metabolic gene expression changes between PKO (y-axis) and rho0 (x-axis) MEFs with respect to TM6 MEFs (calculated as log2 fold-change) (n = 3 biological replicates per line, 12 total). Linear regression lines were fit and Pearson (top value) and Spearman (bottom value) correlation coefficients were calculated with accompanying P values calculated using two-tailed t significance tests. Gene sets were derived from KEGG database metabolic ID MMU00100. (TIF) [file pone.0200925.s003.tif]

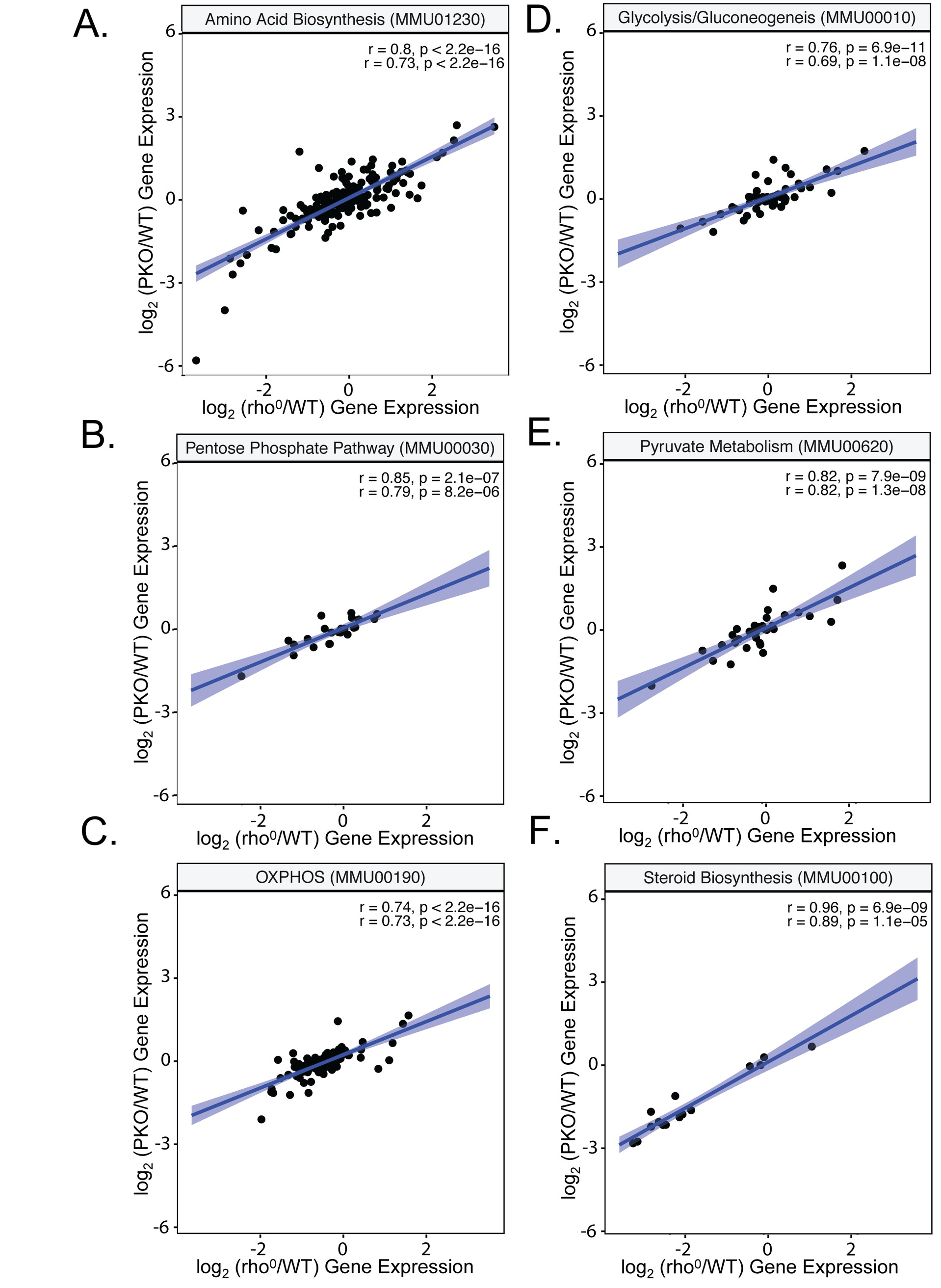

Supplement: S4 Fig — Scatterplot of metabolic gene expression values between PKO (y-axis) and rho0 (x-axis) MEFs with respect to TM6 MEFs (calculated as log2 fold-change) (n = 3 biological replicates per line, 12 total). Linear regression lines were fit and Pearson (top value) and Spearman (bottom value) correlation coefficients were calculated with accompanying significance P values calculated using two-tailed t significance tests. Gene sets were derived from the KEGG database under the identification numbers indicated above each plot. (TIF) [file pone.0200925.s004.tif]

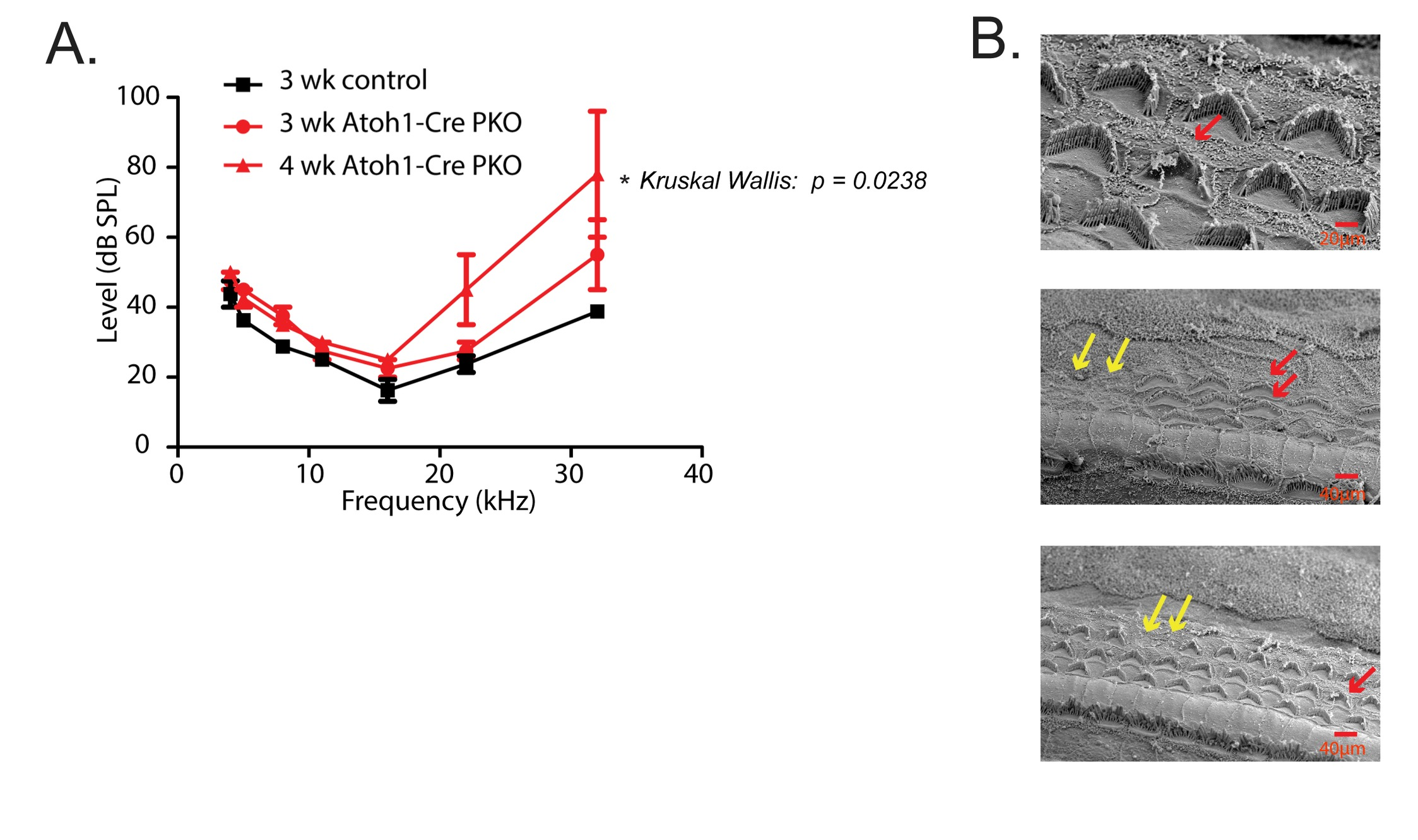

Supplement: S5 Fig — (A) Auditory brainstem response test for WT (black) (n = 3) and Atoh1-Cre PKO mice (red) at 3 weeks (n = 2) and 4 weeks (n = 2), error bars denotes standard error of mean. (B) SEM analysis of hair cell stereocilia (n = 2). Yellow arrows indicate regions that lack cilia, and red arrows indicate regions of stereocilia fusion. (TIF) [file pone.0200925.s005.tif]
